# Documentation status of the modified World Health Organization partograph in public health institutions of Bale zone, Ethiopia

**DOI:** 10.1186/s12978-015-0074-z

**Published:** 2015-09-03

**Authors:** Desalegn Markos, Daniel Bogale

**Affiliations:** Department of Nursing, College of Medicine and Health Sciences, Madawalabu University, P.Box 302, Bale Goba, Ethiopia; Department of Public Health, College of Medicine and Health Sciences, Madawalabu University, P.Box 302, Bale Goba, Ethiopia

## Abstract

**Background:**

Partograph is basically a graphic representation of the events of labour plotted against time in hours. It is designed for early detection of abnormal progress of labour and prevention of prolonged labour in order to reduce risk of Postpartum Haemorrhage (PPH), sepsis, obstructed labour and its sequels such as ruptured uterus and obstetric fistula. However, little is known about documentation status of partograph forms. Therefore, the aim of this study was to assess documentation status of partograph in public health institutions of Bale zone, Ethiopia.

**Methodology:**

Institution based, descriptive cross sectional study based on retrospective document review was conducted to assess partograph forms in public health institutions of Bale zone, from May 1^st^ to June 30, 2014. A total of 508 sampled mothers’ medical records that had been used to monitor labour three months prior to actual data collection period (from February to April 2014) were considered as study populations. The data were analyzed using statistical package for social science version 16.0 and descriptive statistics were computed.

**Results:**

Out of the total 508 mothers’ medical records reviewed, 342 (67.3 %) medical records have partograph forms. Of which, 103 (30.1 %) were not totally recorded and 239 (69.9 %) partograph were recorded only in some manner. From partograph forms recorded in some manner, only 107 (44.8 %) fetal heart rate, 3 (1.3 %) moulding of fetal head, 76 (31.8 %) cervical dilatation, and 17 (7.1 %) descent of head, 57 (23.8 %) uterine contraction, 23 (9.6 %) blood pressure, and 14 (5.9 %) temperature was documented to the standard level.

**Conclusion:**

There is poor documentation of the modified World Health Organization (WHO) partographs during labour in public health institutions of Bale zone, Ethiopia. It is recommended to provide continuous pre-service and on-job training for health care professionals about the standard protocol of partograph documentation and to set policy for each health facility that mandate documentation of the partograph for all laboring mother as per WHO recommendation.

## Introduction

Partograph is a Greek word meaning “Labour Curve”. It comes as a printed one page form on which labour observations are recorded. It provides a graphic overview of the progress of labour and records information about maternal and fetal condition during labour [1]. It also allows early identification and diagnosis of pathological labour. Additionally, its use is critical in preventing maternal and perinatal morbidity and mortality. However, in order to be effective, the partograph must be used correctly [2].

A multicenter trial conducted by World Health Organization (WHO) in Indonesia, Malaysia and Thailand showed that when the partograph was introduced into clinical practice along with a management protocol, labour outcomes were greatly improved. Use of the partograph reduced the number of prolonged labour, the need for augmentation of labour with oxytocin, rates of cesarean section, and the incidence of infection. As a result of this study, WHO recommended that the partograph should be used in monitoring all labour to help identify abnormal progress and women who might need further interventions [3].

Many studies have also reported the practical value of using the partograph, but few have audited its quality in low income countries. A study in Benin showed that appropriate use of partograph was poor [4]. Although midwives recognized that partograph has practical benefits, some of them perceived it to be associated with restrictions in their clinical practice, reduced autonomy, and limited flexibility to treat each woman as an individual [5].

As an additional indication, partograph is a poorly used monitoring tool at the Charlotte Maxeke Johannesburg Hospital in South Africa. It was assumed that inadequate recording of the partograph is one of the factors contributing to maternal mortality and morbidity at this hospital. Observations also showed that health care workers frequently documented the condition of the fetus and the findings on vaginal examination elsewhere in the patient’s obstetric file and managed her accordingly, but omitted to record it in the partograph, which would have been ideal [6].

On the other hand, the study conducted to assess documentation of partograph and its influence on decision making in the University College Hospital (UCH), Ibadan in Nigeria reveled that documentation of partograph was high and not influenced by woman's risk or booking status. Its use significantly influences decision making and associated with positive labour outcome among low/high risk parturient [7].

Despite the WHO recommendation that order partograph should be used to monitor all labour, still it is not widely used in Africa or elsewhere in the developing world. In many countries where it has been mandated without proper training, the partograph serves only as a record of labour (completed after the baby is born) and not as a tool to guide decision making during labour [8]. The federal ministry of health of Ethiopia also recommended the utilization of the new modified WHO partograph during the care given for laboring mother [9]. In Ethiopia, particularly in Bale zone, the documentation status of modified WHO partograph was not studied. Generally, when some of this issue is studied in the country, it is mainly restricted in a very urban area of the country [10–12]. Therefore, the aim of this study was to assess documentation status of the modified WHO partograph in public health institutions of Bale zone, Ethiopia.

## Methodology

### Study area and period

The study was conducted in Public Health institutions of Bale zone from May 1^st^ to June 30, 2014. Bale zone is one of the zones among 18 zone of Oromia regional states located in Southeast of Ethiopia. It is the second largest zone in the region with an area of 67,329.6 km^2^. Robe, the zone city, is located 435 km away from the capital town of Ethiopia; Addis Ababa. The zone contains 20 woredas. The total number of public health institutions in Bale zone is 715. They are composed of 4 hospitals, 76 health centers (H/C), 351 health posts, 179 private clinics, 1 None Governmental Organization (NGO) clinic, 4 other public clinics, 95 pharmacies/drug shop, 1 NGO drug shop and 4 medical drug stores. From these institutions, all health centers and hospitals provide obstetric services [Bale zone Health Office: Annual Health report, (2013) “Unpublished report”].

### Study design

Institution based, descriptive cross sectional study based on retrospective document review was employed to assess partograph forms. Sampled modified WHO partograph that had been used to monitor labour three months prior to actual data collection period (from February to April 2014) were considered as a study population.

### Exclusion criteria

Partographs from mothers’ files having information showing antepartum haemorrhage, intrauterine fetal death (IUFD), and breech presentation was excluded from the study because the documentation of partograph forms is not complete due to contraindication and the nature of the case. For example, vaginal examination is contraindicated for antepartum hemorrhage; as a result information regarding cervical dilatation, moulding, and amniotic fluid (especially whether amniotic membrane is intact or not) cannot be obtained [10].

### Sample size and sampling procedure

The study employed single population proportion sample size determination formula. The proportion (p) of partograph components that was documented according to the standard protocol considered were 32.9 % [10], with 95 % Confidence interval (CI), and 5 % marginal error. Since multistage sampling was considered, the calculated sample size was multiplied by 1.5 for design effect. Then, the final total sample size becomes 508.

### Sampling procedure

From all maternity service provider public health institutions, 44 health institutions were selected by simple random sampling technique. Three consecutive months of the year 2014, namely February, March and April (those three months immediately before the actual data collection period) were selected because of the fact that they provided adequate and current information about partograph documentation in the study area. A total of 508 sampled partograph were allocated to institutions included in the study proportional to the number of the modified WHO partograph used in the maternity units of the respective health institutions. Average numbers of deliveries for the three consecutive months were used in each institution for this purpose. At each study site, systematic sampling were employed to select sampled partograph starting from the last month backwards until the required sample size for each health facility was obtained. This was carried out after calculating sampling fraction for each health institution which was different for each health institution based on the number of delivery reports.

### Data collection tool and procedure

A pretested and structured checklist that was developed after reviewing relevant literatures to the problem under study was used. The checklist was designed to obtain information on the main variables included as components of the modified WHO partograph. In order to produce a more objective assessment, each part of the modified WHO partograph was assessed based on standard protocols. Standard protocols was defined based on the time interval as follows:- (1) cervical dilatation, moulding, descent of head and blood pressure monitored every four hours; (2) fetal heart rate, maternal pulse and uterine contractions monitored every 30 min; (3) Condition of the baby after birth should always be recorded on the card. Based on this standard protocol, documentation status of partograph form was measured as follow; if no information was documented on the parameters of the partograph, it was considered as not recorded, records not meeting any one of the protocol standards or with parts misplaced/missing or inadequate for each parameter of the partograph was judged as substandard, and if all the criteria are met for each parameter on the partograph, the documentation was considered as standard. The condition of the baby such as APGAR score were also assessed and APGAR scores of ≥ 7 were considered satisfactory in this study [10].

Four midwives were recruited as a data collector. All partographs were scrutinized for documentation of cervical dilatation, uterine contraction, fetal heart rate, action line crossed/not crossed, maternal blood pressure, moulding, descent of head, state of membranes and condition of the baby after birth.

### Data quality control

The quality of the data were assured by using validated questionnaire, doing pretest, providing training for data collectors and supervisors, as well as making intensive supervision during data collection. The pretest was done on 5 % of the total sample size in health institution other that actual data collection site. Additionally, content and face validity was checked by another maternal health expert.

### Data processing and analysis

The data were checked for completeness and consistencies. Then, it was cleaned, coded and entered in to computer using statistical package for social sciences (SPSS) windows version 16.0 (SPSS Inc, Chicago, IL, USA). Descriptive statistics were computed to describe documentation status of partograph forms.

### Ethical consideration

The proposal of this research was approved by Ethical Review Committee of Madawalabu University. Furthermore, letter of permission was obtained from Bale zone Health Bureau. Additionally, matrons (head Nurses)/medical directors of each selected health facilities were contacted before the commencement of the study. Besides, the names of clients to whom the documents belonged and name of professionals who completed the partograph forms were not taken.

## Result

Out of the total planned 508 laboring mothers’ medical record, 367 (72.2 %) were reviewed from the Health Centers and 141 (27.8 %) from the Hospitals.

Among the planned mothers’ medical records reviewed, only 342 (67.3 %) medical records have partograph forms. Of which, 103 (30.1 %) were not totally recorded and 239 (69.9 %) forms were recorded only in some manner.

From partograph forms documented in some manner, 107 (44.8 %) fetal heart rate, 3 (1.3 %) moulding of fetal head, 76 (31.8 %) cervical dilatation, 17 (7.1 %) descent of head, 57 (23.8 %) uterine contraction, 23 (9.6 %) blood pressure, and 14 (5.9 %) temperature was documented to the standard level (Table 1).

**Table 1:** Recording of component of maternal and fetal condition of partograph in public health institutions of Bale zone, Ethiopia, May 1^st^ to June 30, 2014

Parameter of labour	Frequency (*n* = 239 except for *)	Percentage
Fetal heart rate		
Not recorded	73	30.5
Substandard	59	24.7
Standard	107	44.8
Was the state of amniotic fluid is recorded appropriately?		
Yes	10	4.2
No	229	95.8
Moulding		
Not recorded	225	94.1
Substandard	11	4.6
Standard	3	1.3
Cervical dilatation		
Not recorded	71	29.7
Substandard	92	38.5
Standard	76	31.8
Alert line crossed^*^ (*n* = 76)		
Yes	18	23.7
No	58	76.3
Action line crossed^*^ (*n* = 76)		
Yes	12	15.8
No	64	84.2
Descent of the head		
Not recorded	189	79.1
Substandard	33	13.8
Standard	17	7.1
Uterine contraction		
Not recorded	79	33.1
Substandard	103	43.1
Standard	57	23.8
Blood pressure		
Not recorded	104	43.5
Substandard	112	46.9
Standard	23	9.6
Temperature recorded		
Not recorded	198	82.8
Substandard	27	11.3
Standard	14	5.9

* indicate for this variable, the denominator used to calculate percentage is n=76

### Fetal condition after delivery

Out of the total 239 partograph forms recorded in some manner, the condition of the baby after births were recorded in 203 (84.9 %) partographs. Among these, 181 (75.7 %) partograph forms showed the presence of spontaneous vaginal delivery (SVD), 192 (80.3 %) alive baby, 133 (55.6 %) implementation of active management of third stages of labour, 189 (79.1 %) documented sex of the newborn and 192 (80.3 %) recorded weight of the newborn. Furthermore, 195 (81.6 %) partographs showed that APGAR score of the newborn was good (Table 2).

**Table 2:** Recording of fetal condition after birth and postnatal information in public health institutions of Bale zone, Ethiopia, May 1^st^ to June 30, 2014

Variable	Frequency	Percentage
Recording condition of the baby after birth		
Yes	203	84.9
No	36	15.1
Mode of delivery		
SVD	181	75.7
C/S	23	9.6
Note recorded	35	14.6
Fetal outcome		
Alive	192	80.3
Dead	3	1.3
Not recorded	44	18.4
Active management of third stage of labour		
Yes	133	55.6
No	106	44.4
Sex of the new born		
Recorded	189	79.1
Not recorded	50	20.9
Weight of the new born		
Recorded	192	80.3
Not recorded	47	19.7
Time of delivery		
Recorded	202	84.5
Not recorded	37	15.5
APGAR score		
Good (7–10)	195	81.6
Not good (1–6)	3	1.3
Not recorded	41	17.2

## Discussion

This study pointed out that there is significant proportion of substandard and not recorded parameters on documenting partograph in public health institutions of Bale zone, Ethiopia, during labour management that may have played a major role in the maternal and fetal health outcomes.

From the total mothers’ medical record reviewed, 342 (67.3 %) medical records have partograph forms. Of which, 103 (20.3 %) were not totally recorded and 239 (69.9 %) were recorded only in some manner. This finding is nearly comparable with the study done in Amhara region [12] where 80 % have partograph paper attached. On the other hand, the finding is against with WHO recommendation which emphasize on monitoring of all labour with the aids partograph [8]. This finding could alarm the need of working hard to change the traditional way of managing labour to contemporary way which is by using simple and inexpensive tool; partograph. It could also inform health facilities managers such as the health centers head and medical director of the hospitals to improve medical records keeping.

The study also revealed that in 73 (30.5 %) partograph forms, fetal heart rates were not totally recorded and the documentation were substandard in 59 (24.7 %) and standard in 107 (44.8) partograph forms. It is nearly comparable with the finding of the study done in Addis Ababa [10] where only 30.7 % of fetal heart rate was recorded as per standard level. But it is not in agreement with the finding of the study done in Amhara region and Dar es Salaam [13] where 79.3 % and 8 % were recorded in standard level, respectively. The reason for the difference of the finding from the study done in Amhara region could be due to the differences in method of assessment where the study conducted in Amhara region was done based on yes/no responses. On the other hand, the disagreement from the study done in Dar es Salaam could be due to the difference in study period where it was done in 2004; ten years back. But, this current study was conducted in a time where much more attention is given for maternal health in the country as well as the world.

The finding further revealed that moulding for the great majority (94.1 %) of partograph forms were not totally recorded, and 11 (4.6 %) partographs were documented to substandard and 3 (1.3 %) to standard level. This finding is comparable with the finding of the study done in Addis Ababa [10] where 86.7 % of moulding were not recorded. It could indicated that health care provider may lack knowledge and skill or they are negligent on assessing moulding of fetal head and necessitate intensive pre service and on job training to fill this gap.

The current study also found that cervical dilatation in 71 (29.7 %) partograph forms were not totally recorded, and 92 (38.5 %) of them were documented to substandard and 76 (31.8 %) to the standard level. It is in nearly similar with the finding of the study done in Addis Ababa, Ethiopia (10) and Uganda [14] where in 32.9 % and 43.9 % of partograph cervical dilatation were documented in standard level. On the other hand, this finding is not comparable with the finding of the study done in Dar es Salaam [13] where only 1.9 % of cervical dilatation was documented as per standard protocol. This could be due to skill difference on the appropriate documentation of partograph on the two areas. In addition, the study period could have its own role for the difference of the study finding; where this current study was done in a time where government and nongovernmental organizations were working hard to meet millennium development goals; reducing maternal child mortality.

The study also reveled that in 189 (79.1 %) partograph forms, descent of fetal head were not totally recorded and were substandard in 33 (13.8 %) and standard in 17 (7.1 %) partograph forms. It is similar with the finding of the study done in Addis Ababa [10] and in UCH, Ibadan [6]. This could implied us that professionals might give less attention in assessing descent of fetal head and as a result their ability to diagnose obstructed labour, which is one of the main function of using partograph forms, is less due to inappropriate documentation of descent of fetal head. But, it is somewhat different from the finding of the study done in Amhara region [12]. This difference could be due to the difference in method of evaluating descent of fetal head on the two studies.

On this study, uterine contraction in 79 (33.1 %), 103 (43.1 %), and 57 (23.8 %) partograph forms were not totally recorded, recorded to substandard and standard level, respectively. This finding is in line with the finding of the study done in Addis Ababa [10]. But it is not in agreement with the finding of the study done in Dar es Salaam [13]. This difference could be due to the great emphasis given for measuring and documenting uterine contraction by professionals in current study area.

The study also revealed that blood pressure in 104 (43.5 %) partograph forms were not totally recorded, and documented to substandard 112 (46.9 %), and standard 23 (9.6 %) level. This finding is in agreement with the study done in Addis Ababa [10]. But it is not consistent with the finding of the study conducted in Dar es Salaam [13]. This difference could be due to the presence of negligence by some professionals in assessing blood pressure and focusing only on expelling out the newborn baby. And since health centers in very remote area of the current study area were included, there might be shortage of vital sign instruments such as blood pressure apparatus in labour ward.

Regarding documentation status of mode of delivery, this study reveled that 181 (75.7 %) partographs revealed spontaneous vaginal delivery, 23 (9.6 %) were with cesarean section and in 35 (14.6 %) it was not recorded. This finding is nearly comparable with the finding of the study done in Amhara region [12] where 63.7 % were delivered in spontaneous and assisted vaginal delivery.

Generally, the overall poor documentation of the partograph found in the present study could reflects the presence of skill gap among health care professionals on how to use partograph forms and poor quality of intrapartum care. It could also indirectly inform us the absence of policy that governs mandatory documentation of partograph or health care professionals might not be aware of this policy.

As a limitation, since the study assessed documentation of partograph during labour through retrospective document review, the documentation may not necessarily reflect the exact extent of use of partograph for monitoring of labour progress at the spot. Besides, the partographs forms might have been used only to record events of labour rather than to guide management of labour which is not seen under this study and need further investigation.

## Conclusion

The study showed that there is poor documentation of the modified WHO partographs in public health institutions of Bale zone, Ethiopia. The findings may implicitly reveal that there is poor management of labour or simply inappropriate documentation of the forms.

It is recommended to support and provide continuous pre-service and periodic on-job training to health professionals on the documentation of partograph, and to conduct regular supportive supervision. Moreover, it is necessary to set a mandatory health facility policy on the documentation of the partograph for all labour as per WHO recommendation. Any interested researcher should attempt further research to assess documentation of partograph by using direct observation method of data collection to avoid report bias as well as to make sure whether the filled partograph forms are filled appropriately or not at the spot.

## References

[1] Kushwah B, Singh AP, Singh S (2013). The Partograph: an Essential Yet underutilized Tool. J Evol. Med. Dent. Sci.

[2] Salama NS, Allah IMA, Heeba MF (2010). The Partograph: Knowledge, Attitude, and Utilization by Professional Birth Attendances in Port-Said and Ismailia. Cities Med J Cairo Univ.

[3] WHO (1994). The application of the WHO partograph in the management of labour. Report of a WHO multicentre study 1990–1991.

[4] Azandegbé N, Testa J, Makoutodé M (2004). Assessment of partogram utilization in Benin. Sante.

[5] Lavender T, Malcolmson L (1999). Is the partogram a help or a hindrance? An exploratory study of midwives’ views. Pract Midwife.

[6] Basu JK, Hoosain S, Leballo G, Leistner E, Masango D, Mercer M (2009). The partogram: A missed opportunity. SAMJ.

[7] Fawole AO, Fadare O (2007). Audit of use of the partograph at the University College Hospital. Ibadan Afr J Med Med Sci.

[8] Maternal and Neonatal Health. The Partograph: An Essential Tool for Decision-Making during Labor. [Internet]. 2005 [cited 2013 Aug 21]. Available from: http://pdf.usaid.gov/pdf_docs/Pnact388.pdf.

[9] Federal Democratic Republic of Ethiopia Ministry of Health. Basic Emergency Obstetric and Newborn Care (BEmONC). 2010.

[10] Yisma E, Dessalegn B, Astatkie A, Fesseha N (2013). Completion of the modified World Health Organization (WHO) partograph during labour in public health institutions of Addis Ababa, Ethiopia. Reprod. Health J.

[11] Yisma E, Dessalegn B, Astatkie A, Fesseha N (2013). Knowledge and utilization of partograph among obstetric care givers in public health institutions of Addis Ababa, Ethiopia. BMC Pregnancy Childbirth.

[12] Abebe F, Birhanu D, Awoke W, Ejigu T (2013). Assessment of knowledge and utilization of the partograph among health professionals in Amhara region, Ethiopia. Sci J Clin. Med.

[13] Nyamtema AS, Urassa DP, Massawe S, Massawe A, Lindmark G, van Roosmalen J (2008). Partogram use in the Dar es Salaam perinatal care study. Int. J. Gynaecol. Obstet..

[14] Ogwang S, Karyabakabo Z, Rutebemberwa E (2009). Assessment of partogram use during labour in Rujumbura Health Sub District, Rukungiri Distric, Uganda. Afr Health Sci.

